# Effect of muscle load tasks with maximal isometric contractions on oxygenation of the trapezius muscle and sympathetic nervous activity in females with chronic neck and shoulder pain

**DOI:** 10.1186/1471-2474-13-146

**Published:** 2012-08-13

**Authors:** Yukiko Shiro, Young-Chang P Arai, Takako Matsubara, Shunsuke Isogai, Takahiro Ushida

**Affiliations:** 1 Department of Physical Therapy, Faculty of Rehabilitation Science, Nagoya Gakuin University, Seto, Japan; 2 Multidisciplinary Pain Centre, Aichi Medical University, School of Medicine, Nagakute, Japan; 3 Department of Rehabilitation, Faculty of Health Sciences, Nihon Fukushi University, Handa, Japan; 4 Department of Rehabilitation, Meitou Hosipital, Nagoya, Japan

**Keywords:** Chronic neck and shoulder pain, Sympathetic nervous activity, Muscle oxygenation

## Abstract

**Background:**

Sympathetic nervous activity contributes to the maintenance of muscle oxygenation. However, patients with chronic pain may suffer from autonomic dysfunction. Furthermore, insufficient muscle oxygenation is observed among workers with chronic neck and shoulder pain. The aim of our study was to investigate how muscle load tasks affect sympathetic nervous activity and changes in oxygenation of the trapezius muscles in subjects with chronic neck and shoulder pain.

**Methods:**

Thirty females were assigned to two groups: a pain group consisting of subjects with chronic neck and shoulder pain and a control group consisting of asymptomatic subjects. The participants performed three sets of isometric exercise in an upright position; they contracted their trapezius muscles with maximum effort and let the muscles relax (Relax). Autonomic nervous activity and oxygenation of the trapezius muscles were measured by heart rate variability (HRV) and Near-Infrared Spectroscopy.

**Results:**

Oxyhemoglobin and total hemoglobin of the trapezius muscles in the pain group were lower during the Relax period compared with the control group. In addition, the low frequency / high frequency (LF/HF) ratio of HRV significantly increased during isometric exercise in the control group, whereas there were no significant changes in the pain group.

**Conclusions:**

Subjects with neck and shoulder pain showed lower oxygenation and blood flow of the trapezius muscles responding to isometric exercise, compared with asymptomatic subjects. Subjects with neck and shoulder pain also showed no significant changes in the LF/HF ratio of HRV responding to isometric exercise, which would imply a reduction in sympathetic nervous activity.

## Background

Chronic neck and shoulder pain is very common symptom especially in females. A systematic review reports that arm force, arm posture, duration of sitting, work place design [1], repetitive hand and finger movements and monotonous work task [2] cause neck and shoulder disorders. In particular, a correlation has been identified between neck and shoulder pain and the trapezius muscle [3,4]. The trapezius muscle is well capillarized [4,5]. Most hypotheses for the development and maintenance of work-related muscle pain propose that muscle-cell activity produces energy demands that are not met by circulation, thereby resulting in hypoxia, energy crisis, and accumulation of metabolites in the muscle [6]. Several studies showed that metabolic insufficiencies are related to pain perception of workers with trapezius myalgia [7,8]. Furthermore, impaired regulation of microcirculation occurs in the trapezius muscle in cases of chronic neck pain [4,5] and insufficient muscle blood flow and oxygenation have been observed among workers with chronic neck and shoulder complaints [7,8]. In contrast, activation of skeletal muscle fibers by somatic nerves leads to vasodilation. A previous study showed that low-level static contraction did increase trapezius muscle blood flux [9]. A lot of the results in an association between muscle activity and pain are not consistent. The pathogenic mechanisms of chronic neck and shoulder pain development are likely to be multifactorial.

Sympathetic nerve activity contributes to vasoconstriction and the maintenance of arterial blood pressure [10]. However, several studies have demonstrated autonomic dysfunction in chronic pain syndromes. Impaired function of sympathetic nerves was observed in patients with complex regional pain syndrome (CRPS) [11]. Furthermore, patients with fibromyalgia showed autonomic dysfunction characterized by persistent autonomic nervous system hyperactivity at rest and hypo-reactivity during stress [12]. Thus, we speculate that there may be autonomic dysfunction in subjects with chronic neck and shoulder pain.

Heart rate variability (HRV) has been used as a biomarker of autonomic nervous system function. HRV is a reliable method to obtain information on sympathetic and parasympathetic contributions to heart rate, and several studies have shown that pain increases sympathetic activity [13]. Frequency fluctuations of HRV in the range of 0.04-0.15 Hz (low frequency, LF) are considered to be markers of sympathetic and parasympathetic nerve activity, and high frequency (HF) fluctuations in the range of 0.15-0.4 Hz are considered markers of parasympathetic nerve activity. Thus, the LF/HF ratio is considered to be an index of sympathetic nerve activity [13,14].

We hypothesized that subjects with chronic neck and shoulder pain would have autonomic dysfunction to muscle load, thereby leading to insufficient muscle blood flow and oxygenation of the trapezius muscle. The aim of the present study was to see how heart rate variability and oxygenation of the trapezius muscle respond to muscle load tasks with maximal isometric contraction in subjects with chronic neck and shoulder pain.

## Methods

After receiving approval from the Nagoya Gakuin University Board of Ethics and obtaining written informed consent, 30 female participants were recruited for the present study. Exclusion criteria were serious conditions such as previous trauma to the neck or shoulder, cardiovascular or neurological disease, diabetes, menstruation, or administration of sedatives, analgesics, or other medication. Participants were assessed on pain intensity using a verbal rating scale (VRS) and visual analogue scale (VAS), pain-related disability using Neck Disability Index (NDI). For the VRS, the intensity of neck pain was rated on a numerical scale from 0 to 3 (0 = no pain, 1 = mild pain, 2 = moderate pain, and 3 = severe pain). For the VAS, the pain intensity was assessed using a horizontal 100-mm line with the words “no pain” at one end and “worst pain imaginable” at the other. The participants were assigned into two groups; a pain group consisting of subjects who scored higher than 1 on VRS and more than 10 mm on VAS, the control group consisting of subjects who answered 0 for both VRS and VAS. Subsequently, a clinical neck and shoulder examination was performed with the pain group. After a positive clinical diagnosis of tightness of the upper trapezius muscle and palpable tenderness of the upper trapezius muscle was confirmed, the patient was assigned to the pain group. In the present study, ultimately 14 females qualified for the pain group and 12 females qualified for the control group (Table 1). The pain group subjects complained of the pain bilaterally, although there was laterality for pain intensity.

**Table 1:** Characteristics of the control group and the pain group

	**Control group (n = 12)**		**Pain group (n = 14)**				***P*value**
Age (yr)	28.7	(4.6)	29.5		(4.1)		0.625
Height (cm)	157.3	(3.8)	157.9		(5.7)		0.898
Weight (kg)	49.3	(6.2)	50.9		(9.4)		0.939
BMI	20.0	(1.9)	20.5		(3.6)		0.430
VRS	0	(0)	Rt. 1.8	(0.7)	Lt. 1.7	(0.8)	-
VAS (mm)	0	(0)	Rt. 44.7	(23.2)	Lt. 44.0	(21.2)	-
NDI	1.5	(1.6)	5.8		(4.8)		0.006

Values expressed as mean (SD). BMI: body mass index, VRS: verbal rating scale, VAS: visual analogue scale, NDI: neck disability index.

All measurements were performed in the afternoon. Trapezius muscles oxygenation were measured using Near-Infrared Spectroscopy (NIRS) (NIRO 200, Hamamatsu Photonics, Japan) bilaterally. Probes were placed at the transverse section on both sides of the upper trapezius muscles at the midpoint between the spinous process of the seventh cervical vertebrae and the acrominon. Measurements were given as concentration change in μM of oxyhemoglobin (ΔO_2_Hb), deoxyhemoglobin (ΔHHb), and total hemoglobin (ΔTHb = ΔO_2_Hb + ΔHHb) from baseline [15]. The electrocardiogram (ECG) signals were obtained from a portable ECG recorder (AC301A, GMS, Tokyo, Japan) and transferred to a computer loaded with HRV analysis software (TARAWA/WIN; Suwa Trust, Tokyo, Japan). The R-R intervals (RRIs) were obtained every 10 seconds. The two components of power of the RRI (ms.ms), LF (0.04-0.15 Hz) and HF (0.15-0.4 Hz), were calculated. The participants were allowed to sit comfortably on a chair in a quiet environment for 10 minutes. Then, the record of the trapezius muscles oxygenation and the ECG signal for heart rate variability (HRV) analysis started. After a five-minute measurement of the first rest period, the participants were instructed to perform three sets of isometric exercise in an upright position; while wearing wrist weights (2 kg on each side), they contracted their bilateral trapezius muscles with maximum effort (MAX) for 1 minute and let the muscles relax (Relax) for 2 minutes. Participants were directed, where necessary to make more effort of contraction. Participants were coached to use only the trapezius muscles without extending their neck and abducting their shoulder and flexing their elbow. After three sets of isometric exercise, the participants were observed for another 5 minutes while sitting (the second rest).

For data evaluation, the areas under the curve (AUC) of NIRS values and LF/HF ratio were measured for the first rest period, each of three sets of MAX and Relax period, and the second rest period [16]. Data were presented as mean (SE). Data were analyzed using Mann–Whitney test or Friedman test, where appropriate. After Friedman test for repeated measure analysis, *post hoc* multiple comparison tests were performed with Tukey. A *p* value < 0.05 was considered statistically significant.

## Results

There were no significant differences in age, height, and weight between the two groups (Table 1). NDI was higher in the pain group than in the control group (Table 1).

**Figure 1:**
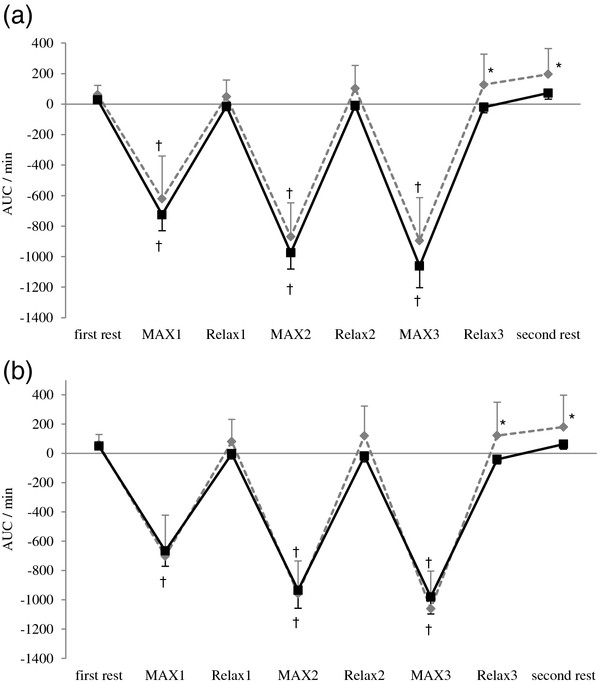
**Change in oxyhaemoglobin (ΔO_2_Hb) at: (a) the right and (b) the left trapezius muscle.** AUC: area under the curve. MAX 1~MAX 3: the trapezius muscles contract with maximum effort for 1 min. Relax 1~Relax 3: the trapezius muscles relax for 2 min. Values are presented as mean, SE. †, different from the ΔO_2_Hb first rest (p < 0.01). *, different from the ΔO_2_Hb of the control group (p < 0.05).

**Figure 2:**
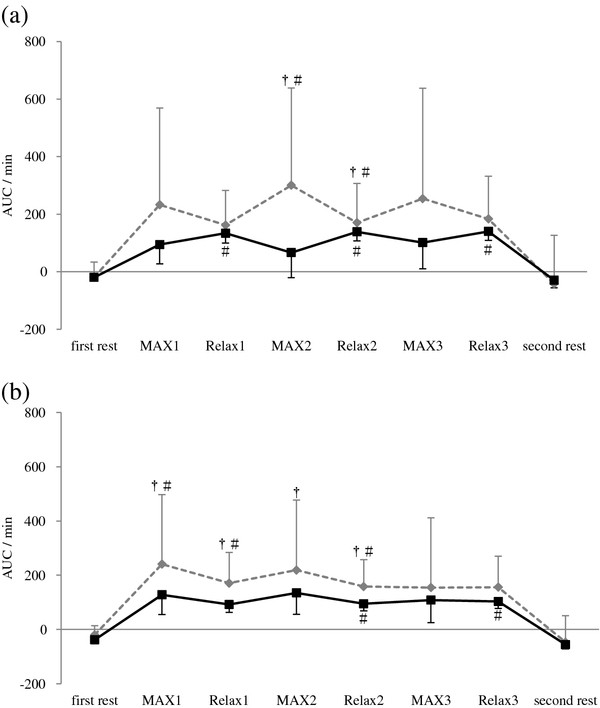
**Change in deoxyhemoglobin (ΔHHb) at: (a) the right and (b) the left trapezius muscle.** AUC: area under the curve. MAX 1~MAX 3: the trapezius muscles contract with maximum effort for 1 min. Relax 1~Relax 3: the trapezius muscles relax for 2 min . Values are presented as mean, SE. †, different from the ΔHHb first rest (p < 0.01). #, different from the ΔHHb second rest (p < 0.05).

**Figure 3:**
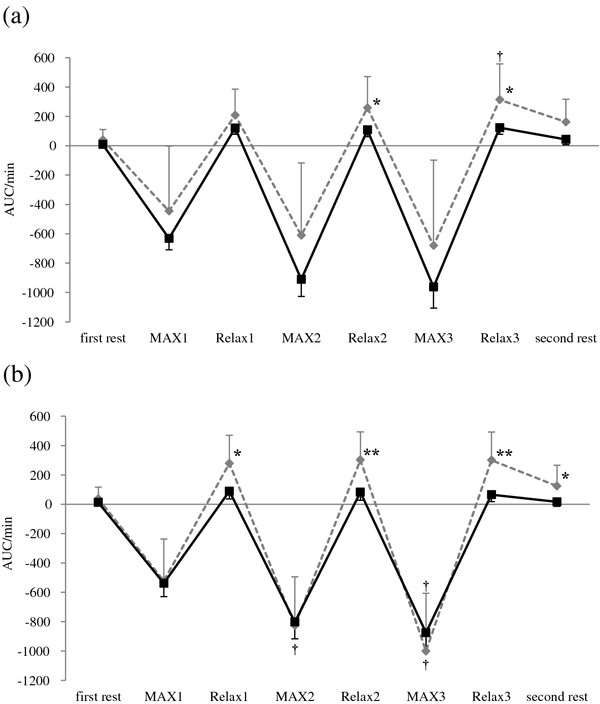
**Change in total hemoglobin (ΔTHb) at: (a) the right and (b) the left trapezius muscle.** AUC: area under the curve. MAX 1~MAX 3: the trapezius muscles contract with maximum effort for 1 min. Relax 1~Relax 3: the trapezius muscles relax for 2 min. Values are presented as mean, SE. †, different from the ΔTHb first rest (p < 0.05). *, **, different from the ΔTHb of the control group (p < 0.05, p < 0.01).

**Figure 4:**
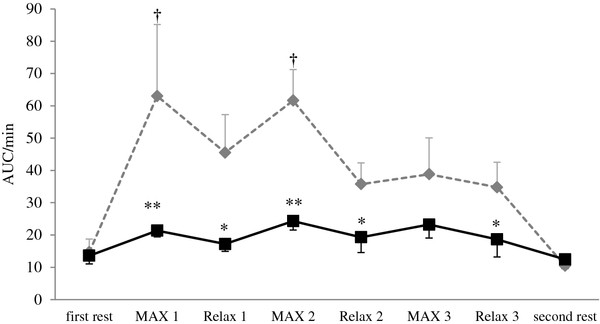
**Change in the LF/HF ratio of heart rate variability.** AUC: area under the curve. MAX 1~MAX 3: the trapezius muscles contract with maximum effort for 1 min. Relax 1~Relax 3: the trapezius muscles relax for 2 min . Values are presented as mean, SE. †, different from the LF/HF ratio first rest (p < 0.01). *, **, different from the LF/HF ratio of the control group (p < 0.05, p < 0.01).

ΔO_2_Hb significantly decreased during each MAX period compared with the first rest period, and recovered to the level of the first rest period during each Relax period in both groups. However, ΔO_2_Hb in the pain group was lower during Relax 3 and second rest periods compared with the control group (Figure 1). ΔHHb significantly increased during isometric exercise period compared with the first rest period and the second rest period in both groups, with no significant difference between groups (Figure 2). ΔTHb in the pain group was lower during Relax 2 and Relax 3 periods at the right trapezius muscle and each Relax and second rest periods at the left trapezius muscle compared with the control group (Figure 3). The LF/HF ratio significantly increased during the first and second MAX periods compared with the first rest period in the control group. In contrast, the pain group induced no significant changes in the LF/HF ratio of HRV responding to isometric exercise. Furthermore, the LF/HF ratio in the pain group was lower during MAX and Relax period compared with the control group (Figure 4).

## Discussion

This study showed that ΔO_2_Hb significantly decreased during each MAX period, but ΔHHb significantly increased during isometric exercise period in both groups, with no significant difference between the groups. ΔO_2_Hb in the pain group was lower during Relax 3 and second rest period and ΔTHb in the pain group was lower during each Relax period compared with the control group. Although the LF/HF ratio of HRV significantly increased after isometric exercise in the control group, there were no significant changes in the pain group.

In the present study, ΔO_2_Hb and ΔTHb in the pain group were lower during Relax period compared with those of the control group. Subjects with neck pain and low back pain exhibited a reduced aerobic capacity of the trapezius and erector spinate muscles [17,18]. If the aerobic capacity of the muscle is impaired, the recovery time of muscle oxygenation is prolonged [18]. We thus postulated that in the present study subjects with chronic neck and shoulder pain had reduced aerobic capacity of the trapezius muscles, thereby leading to an insufficient recovery of oxygenation of the muscles during each Relax period.

Chronic neck and shoulder pain is believed to develop in response to prolonged muscle activity causing metabolic disturbances. A previous study showed that chronic neck pain increased amplitude of the root mean squared electromyography (rms-EMG) of trapezius muscles during muscle contraction and rest as well, which would indicate increased basic muscle tension secondary to the impaired muscle microcirculation [19]. However, shoulder and neck pain occur commonly during work involving very low levels of muscle activity. Also the low level contraction like the work task increased the trapezius muscle blood flux [3,9]. Furthermore, subjects with neck pain did not differ from the healthy subjects in the EMG levels of the trapezius muscles during the work task [3]. Therefore, muscle activity and the relation of oxygenation are not clear with chronic neck and shoulder pain.

On the other hand, the blood flow in the exercising muscle is regulated by not only the activity of the somatic but also sympathetic nervous system [10,20]. An increase in sympathetic nerve activity exerts extrinsic control over the skeletal muscle vasculature, through the release of norepinephrine to cause vasoconstriction [10,21]. Simultaneously, increased somatomotor nerve activity causes contraction of skeletal muscle fibers, thereby relaxing vascular smooth muscle cells to increase capillary perfusion and vascular conductance [10]. In addition, the ability of arterioles to surpass sympathetic vasoconstriction is enhanced in contracting muscles with ‘functional sympatholysis’ promoting an increase in blood flow to active muscle fibers [10,21,22]. Moreover, sympathetic cholinergic nerve contributes to increased muscle blood flow at the onset of static exercise in cats [23]. The existence of the sympathetic cholinergic mechanism in humans has been suggested, because emotional stress increases skeletal muscle blood flow, and the increase in skeletal muscle blood flow is blocked by either atropine or sympathectomy [23]. However, several studies showed that the abnormality in the sympathetic nervous system might generate and sustain chronic pain [11,24].

In the present study, subjects with neck and shoulder pain induced no significant changes in the LF/HF ratio of HRV responding to isometric exercise. Hence, the sympathetic nervous system of the subjects with neck and shoulder pain was an abnormality. We therefore believe that insufficient muscle blood flow and oxygenation of the trapezius muscles might derive from the lower sympathetic nerve response during isometric exercise among subjects with neck and shoulder pain compared with asymptomatic subjects.

Jinbo et al. measured the recovery time of trapezius muscle oxygenation in subjects with neck pain after performing one set of isometric exercise and they found that the recovery time was prolonged [18]. However, we did not find a prolonged recovery time in a pilot study using one set of isometric exercise. Static and high repetitive work tasks have been identified as a risk factor for work-related trapezius myalgia [25,26]. And a previous study reported increased intramuscular lactate and glutamate in the trapezius muscle responding to repetitive work [8]. Also, oxyhemoglobin of the trapezius muscle decreased during repeated work in subjects with and without trapezius myalgia, but only subjects without myalgia recovered back to the baseline level during a subsequent recovery period. In contrast, no such recovery occurred in subjects with myalgia [7]. We thus postulated that repeated isometric contraction of the trapezius muscles could easily influence muscle oxygenation especially in subjects with chronic neck and shoulder pain, and thereby we used a muscle load task with three-time repeated isometric exercises in the present study.

There is a limitation to present study. We did not measure EMG with trapezius muscles. We need an evaluation of the relation between trapezius muscle activity and oxygenation.

## Conclusion

ΔO_2_Hb and ΔTHb in subjects with neck and shoulder pain were lower during each Relax period compared with those of the control group. Furthermore, the subjects with neck and shoulder pain induced no significant changes in the LF/HF ratio of HRV responding to isometric exercise.

## Competing interests

The authors declare that they have no competing interests.

## Authors’ contribution

YS conceived of the study, participated in its study, and conducted all experiments. YPA, TM and SI conducted the acquisition of data and performed the statistical analysis. YAP and TU helped to draft the manuscript. All authors read and approved the final manuscript.

## Pre-publication history

The pre-publication history for this paper can be accessed here:

http://www.biomedcentral.com/1471-2474/13/146/prepub
